# Supplementary material for: Global patterns of potential future plant diversity hidden in soil seed banks
Source: Nat Commun. 2021 Dec 2;12:7023. doi: 10.1038/s41467-021-27379-1 (PMC8639999; doi:10.1038/s41467-021-27379-1)
Supplement: Supplementary file 1 — Supplementary Information [file 41467_2021_27379_MOESM1_ESM.pdf]

Supplementary Information:
Global patterns of potential future plant diversity hidden in soil seed banks

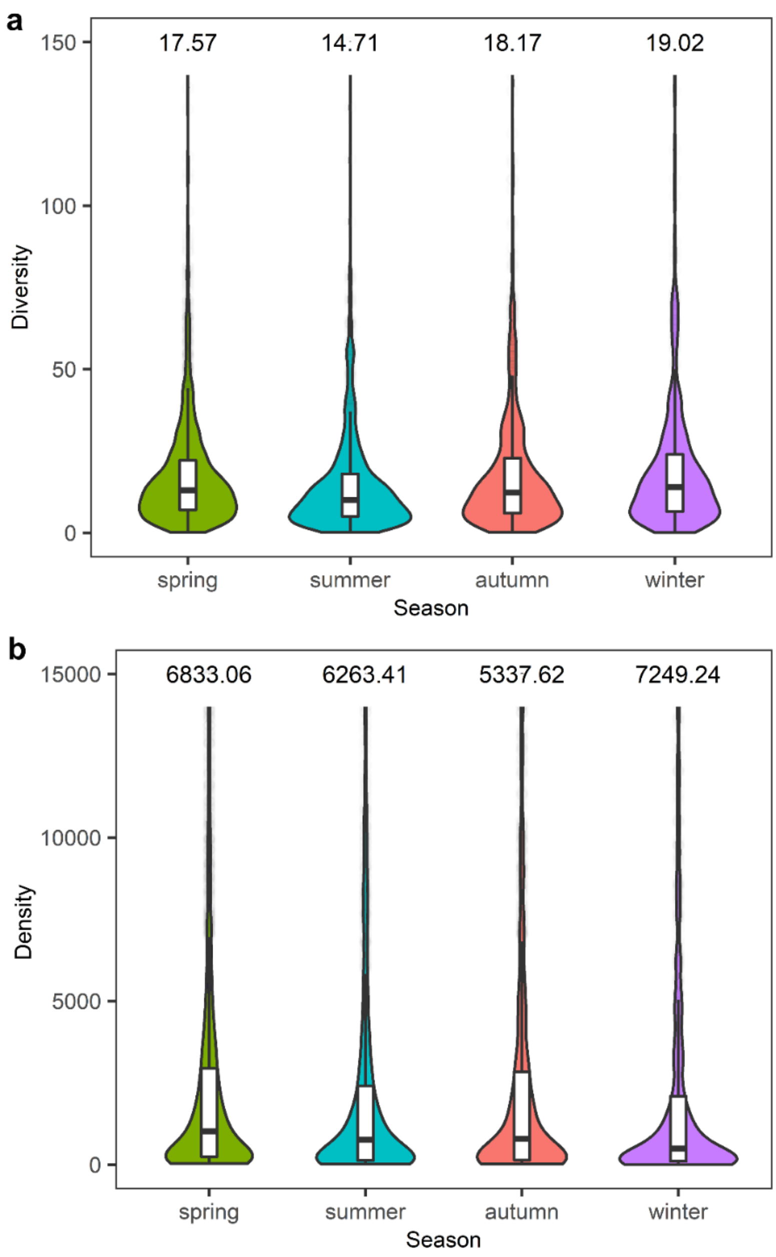

Supplementary Figure 1. Soil seed bank (a) diversity in terms of number of species and (b) density as number of seeds per m^2 in different seasons. The black lines represent the median. The lower and upper hinges correspond to the 25th and 75th percentiles. Mean values are shown at the top of each column. Sample size: n=6480 in (a) and 9218 in (b).

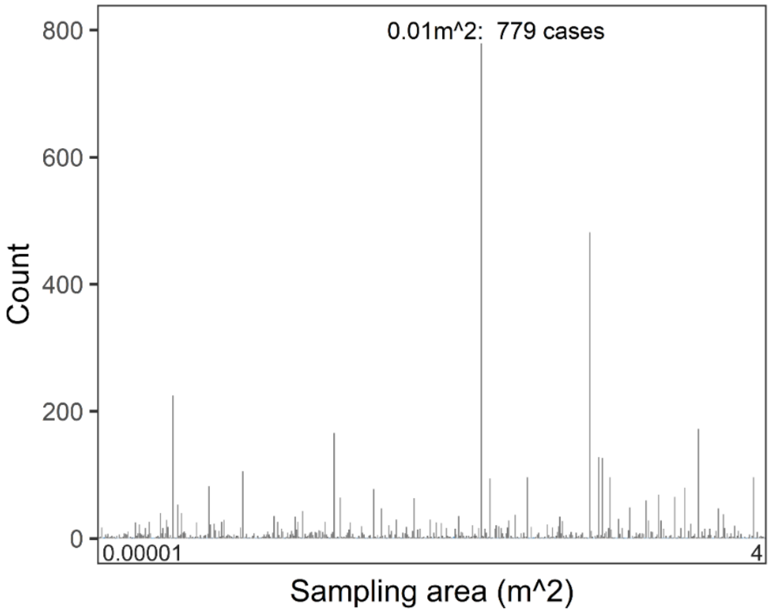

Supplementary Figure 2. Frequency of sampling area in the studies on soil seed bank diversity. The most commonly reported sampling area is shown above the column.

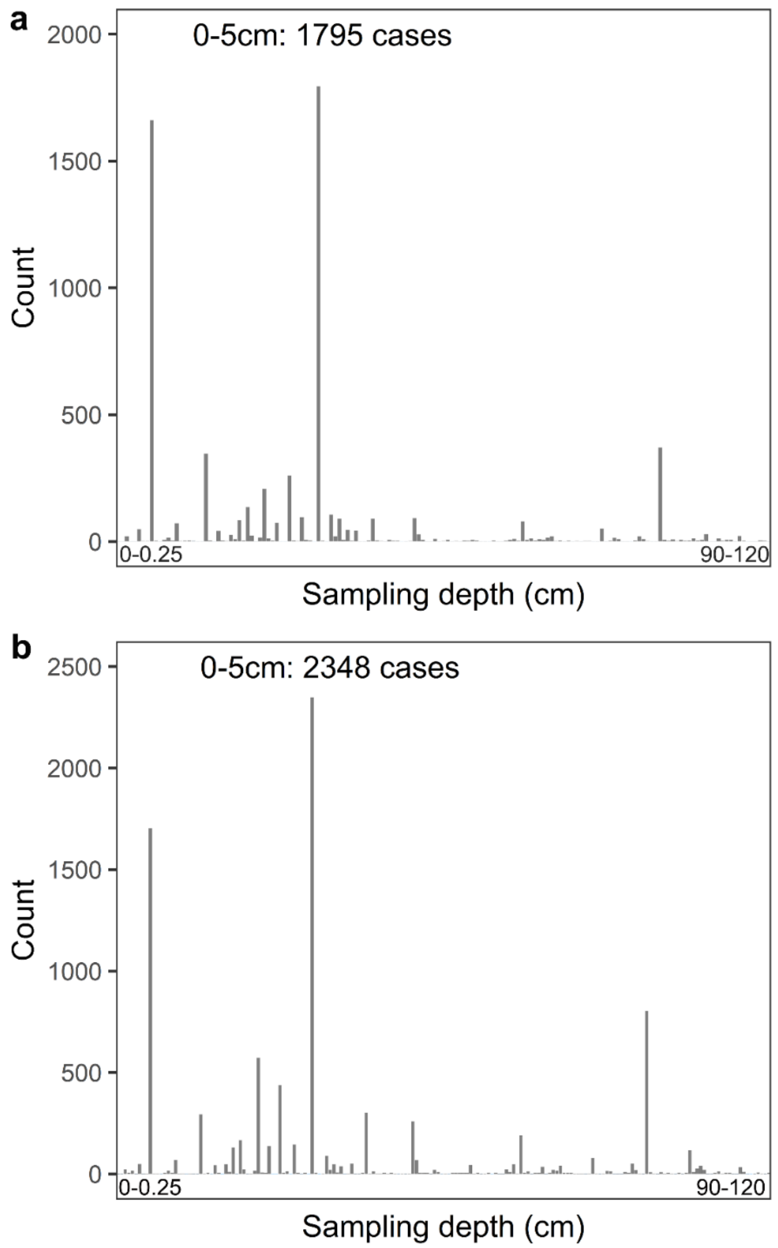

Supplementary Figure 3. Frequency of sampling depth in the studies on soil seed bank (a) diversity and (b) density. The most commonly reported sampling depth is shown above the column.

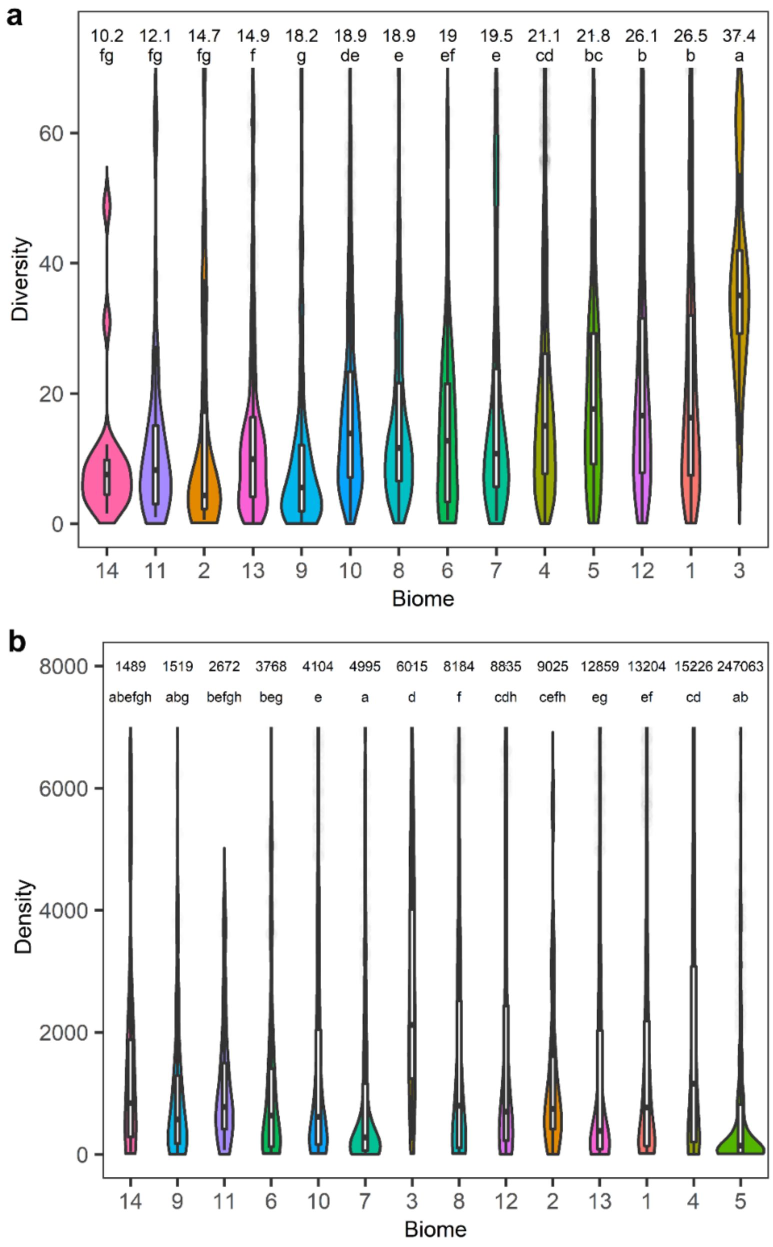

Supplementary Figure 4. Comparison of soil seed bank (a) diversity and (b) density among biomes. In the violin plots, the black lines in the white bars are the median values, the thick black bar the interquartile range and thin line extending from the white bar the upper (max) and lower (min) adjacent values in the data. Mean values are shown at the top of each column. Different letters indicate significant differences. 1, tropical & subtropical moist broadleaf forests; 2, tropical & subtropical dry broadleaf forests; 3, tropical & subtropical coniferous forests; 4, temperate broadleaf & mixed forests; 5, temperate conifer forests; 6, boreal forests/taiga; 7, tropical & subtropical grasslands, savannas & shrublands; 8, temperate grasslands, savannas & shrublands; 9, flooded grasslands & savannas; 10, montane grasslands & shrublands; 11, tundra; 12, Mediterranean forests, woodlands & scrub; 13, deserts & xeric shrublands; 14, mangroves. Sample size: n=6480 in (a) and 9218 in (b).

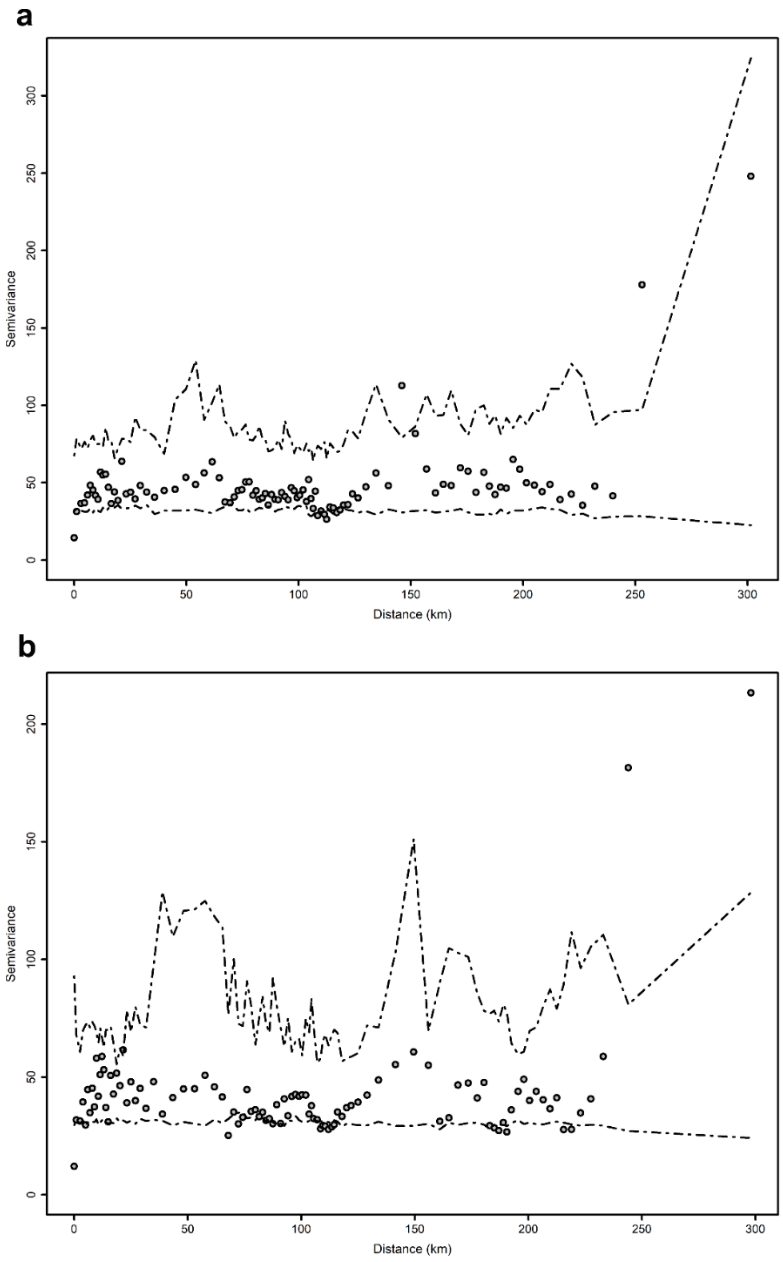

Supplementary Figure 5. Semivariograms showing spatial autocorrelation in the data of soil seed bank (a) diversity and (b) density. Dashed lines are the envelop of semivariance obtained by permutation.

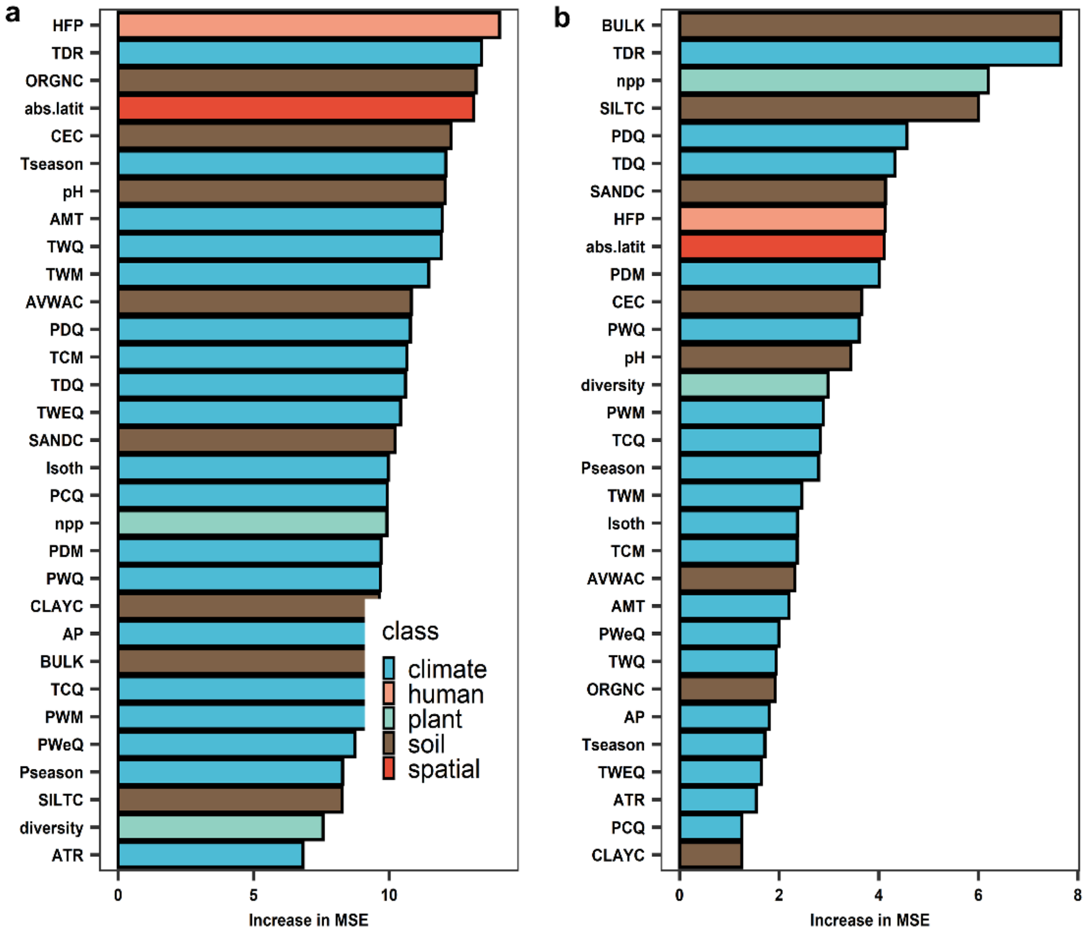

Supplementary Figure 6. Percentage increase in mean square error (% inc. MSE) of random forests run with all 31 predictors. (a) Soil seed bank diversity; (b) Density. See Table S1 for abbreviations of predictors on the y-axis.

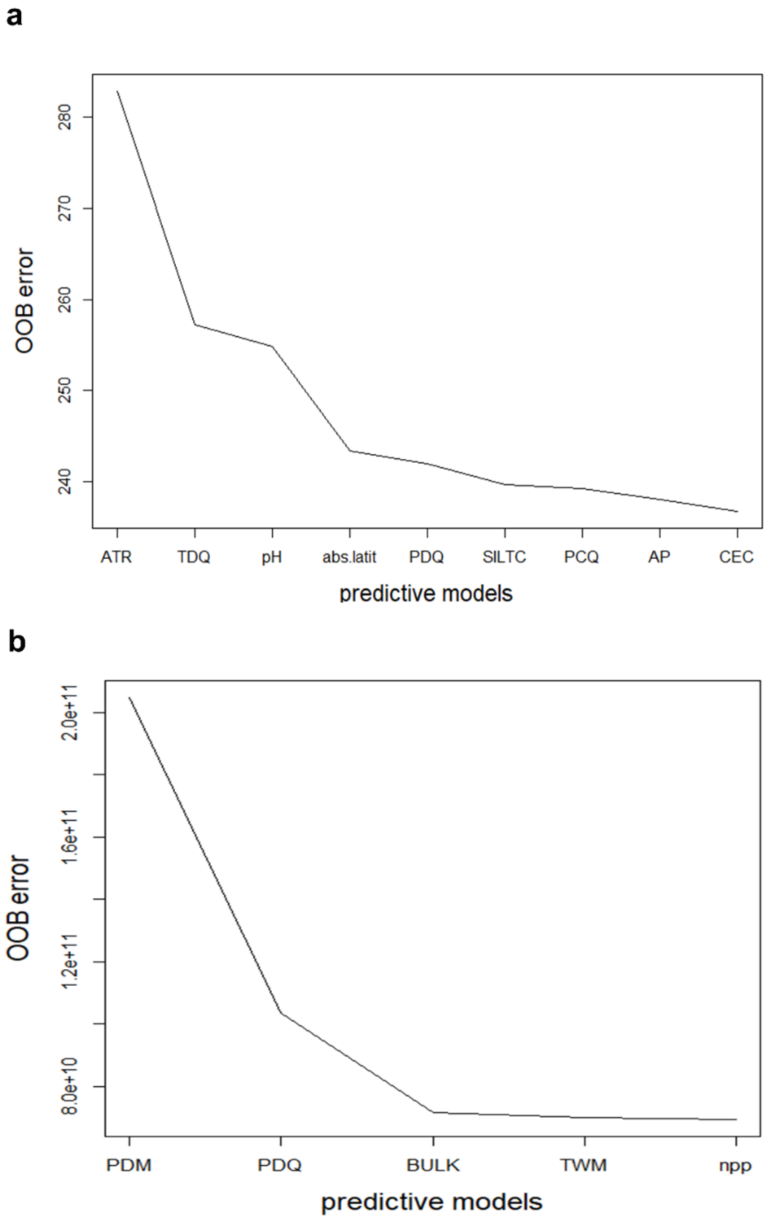

Supplementary Figure 7. Variable selection using random forests. (a) Soil seed bank diversity; (b) Density. OOB error, the out-of-bag error. See Table S1 for abbreviations of predictors on the x-axis.

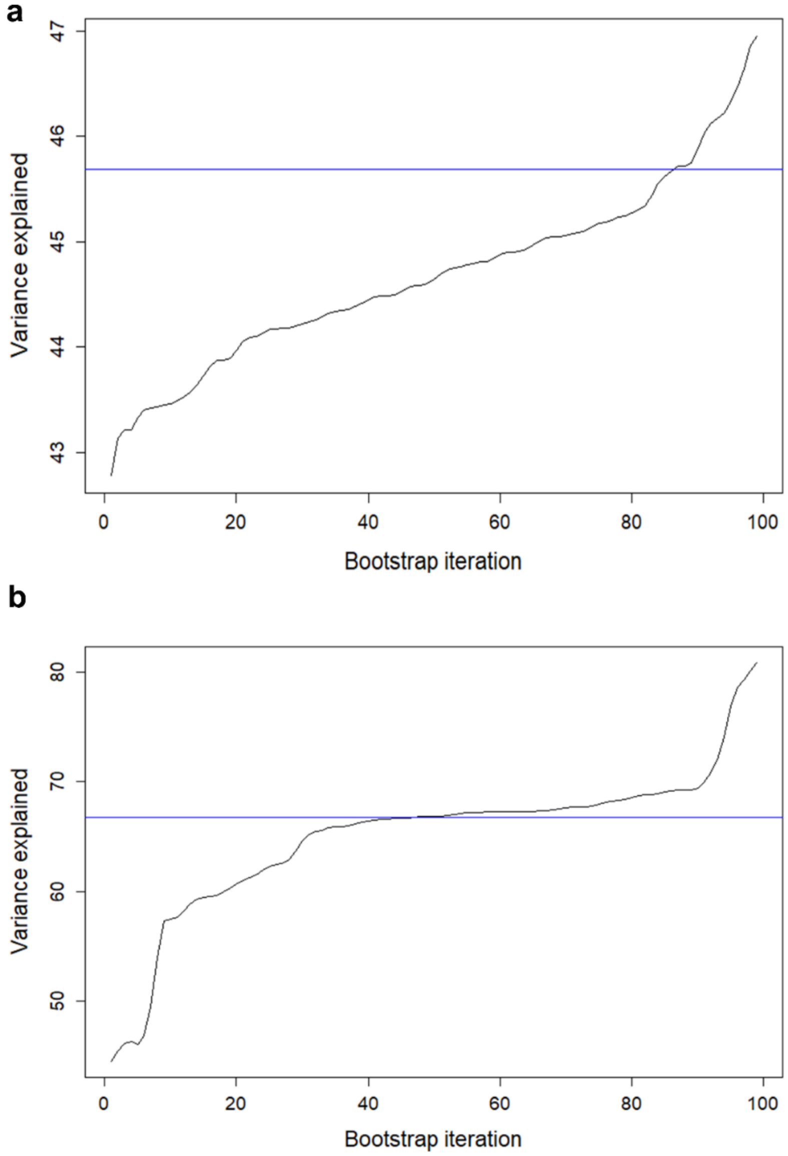

Supplementary Figure 8. Cross validation of final random forest models (with the most important predictors). (a) Soil seed bank diversity; (b) Density.

Supplementary Table 1. The relationship between soil seed bank diversity and sampling area. Linear regressions were used to analyzed the relationships. The parameters for regression at the log scale are shown.

Code	Biome	Intercept	Slope	P-value
1	Tropical & Subtropical Moist Broadleaf Forests	1.114	-0.031	0.155
2	Tropical & Subtropical Dry Broadleaf Forests	0.865	0.044	0.595
3	Tropical & Subtropical Coniferous Forests	1.459	-0.028	0.818
4	Temperate Broadleaf & Mixed Forests	1.204	0.055	0
5	Temperate Conifer Forests	1.26	0.124	0.091
6	Boreal Forests/Taiga	0.282	-0.362	0
7	Tropical & Subtropical Grasslands, Savannas & Shrublands	0.95	-0.043	0.202
8	Temperate Grasslands, Savannas & Shrublands	1.175	0.096	0
9	Flooded Grasslands & Savannas	1.376	0.344	0
10	Montane Grasslands & Shrublands	1.129	0.015	0.482
11	Tundra	0.836	-0.07	0.321
12	Mediterranean Forests, Woodlands & Scrub	1.12	-0.023	0.495
13	Deserts & Xeric Shrublands	0.862	-0.073	0.011
14	Mangroves	1.021	0.159	0.504

Supplementary Table 2. The relationships between soil seed bank (a) diversity and (b) density and sampling depths (upper and lower boundaries of sampling soil depths/slices). Linear regressions were used to analyzed the relationships.

Code	Biome	Intercept	Slope.upper	Slope.lower	P-value
	**(a) diversity**				
1	Tropical & Subtropical Moist Broadleaf Forests	0.829	-0.06	0.04	0.126
2	Tropical & Subtropical Dry Broadleaf Forests	-0.157	-0.126	0.471	0.242
3	Tropical & Subtropical Coniferous Forests	1.695	0.01	-0.139	0.066
4	Temperate Broadleaf & Mixed Forests	0.95	-0.033	0.004	0.039
5	Temperate Conifer Forests	1.198	0.015	-0.041	0.003
6	Boreal Forests/Taiga	0.749	-0.062	-0.031	0.12
7	Tropical & Subtropical Grasslands, Savannas & Shrublands	0.753	-0.06	-0.023	0.155
8	Temperate Grasslands, Savannas & Shrublands	0.776	-0.059	-0.076	0.152
9	Flooded Grasslands & Savannas	-0.674	-0.15	0.927	0.312
10	Montane Grasslands & Shrublands	1.045	-0.011	0.018	0.008
11	Tundra	0.404	-0.097	0.066	0.447
12	Mediterranean Forests, Woodlands & Scrub	0.951	-0.041	-0.015	0.031
13	Deserts & Xeric Shrublands	1.024	0.021	0.056	0.016
14	Mangroves	0.354	-	0.626	0.081
	**(b) density**				
1	Tropical & Subtropical Moist Broadleaf Forests	1.84	-0.196	-0.073	0.147
2	Tropical & Subtropical Dry Broadleaf Forests	4.617	0.002	-2.219	0.349
3	Tropical & Subtropical Coniferous Forests	4.333	-0.008	-0.987	0.359
4	Temperate Broadleaf & Mixed Forests	2.378	-0.144	0.154	0.12
5	Temperate Conifer Forests	0.279	-0.39	0.106	0.232
6	Boreal Forests/Taiga	3.488	0.033	-0.62	0.032

7	Tropical & Subtropical Grasslands, Savannas & Shrublands	1.862	-0.13	0.076	0.115
8	Temperate Grasslands, Savannas & Shrublands	2.539	-0.105	-0.243	0.1
9	Flooded Grasslands & Savannas	1.691	-0.025	1.108	0.083
10	Montane Grasslands & Shrublands	2.016	-0.146	0.073	0.096
11	Tundra	4.21	0.216	-0.012	0.292
12	Mediterranean Forests, Woodlands & Scrub	3.456	0	-0.24	0.012
13	Deserts & Xeric Shrublands	1.847	-0.171	0.03	0.114
14	Mangroves	2.166	-	0.871	0.055

Supplementary Table 3. Explanation, source, and resolution of the 31 predictors in this study.

Number	Class	abbreviation	Explanation	Source	resolution
1	climate	AMT	Annual Mean Temperature	WorldClim v.2[1]	5 arc-min
2	climate	T_{DR}	Mean Diurnal Range (Mean of monthly (max temp - min temp))	WorldClim v.2[1]	5 arc-min
3	climate	Isoth	Isothermality (#2/#7) (* 100)	WorldClim v.2[1]	5 arc-min
4	climate	T_{season}	Temperature Seasonality (standard deviation *100)	WorldClim v.2[1]	5 arc-min
5	climate	T_{WM}	Max Temperature of Warmest Month	WorldClim v.2[1]	5 arc-min
6	climate	T_{CM}	Min Temperature of Coldest Month	WorldClim v.2[1]	5 arc-min
7	climate	ATR	Annual Temperature Range (#5-#6)	WorldClim v.2[1]	5 arc-min
8	climate	T_{WEQ}	Mean Temperature of Wettest Quarter	WorldClim v.2[1]	5 arc-min
9	climate	T_{DQ}	Mean Temperature of Driest Quarter	WorldClim v.2[1]	5 arc-min
10	climate	T_{WQ}	Mean Temperature of Warmest Quarter	WorldClim v.2[1]	5 arc-min
11	climate	T_{CQ}	Mean	WorldClim v.2[1]	5 arc-min

			Temperature of Coldest Quarter		
12	climate	AP	Annual Precipitation	WorldClim v.2[1]	5 arc-min
13	climate	P_{WM}	Precipitation of Wettest Month	WorldClim v.2[1]	5 arc-min
14	climate	P_{DM}	Precipitation of Driest Month	WorldClim v.2[1]	5 arc-min
15	climate	P_{season}	Precipitation Seasonality (Coefficient of Variation)	WorldClim v.2[1]	5 arc-min
16	climate	P_{WeQ}	Precipitation of Wettest Quarter	WorldClim v.2[1]	5 arc-min
17	climate	P_{DQ}	Precipitation of Driest Quarter	WorldClim v.2[1]	5 arc-min
18	climate	P_{WQ}	Precipitation of Warmest Quarter	WorldClim v.2[1]	5 arc-min
19	climate	P_{CQ}	Precipitation of Coldest Quarter	WorldClim v.2[1]	5 arc-min
20	soil	BULK	Bulk density	SoilGrids250m[2]	250 m
21	soil	CEC	Cation exchange capacity	SoilGrids250m [2]	250 m
22	soil	CLAYC	Clay (mass %)	SoilGrids250m[2]	250 m
23	soil	ORGNC	Organic carbon content	SoilGrids250m[2]	250 m
24	soil	pH	pH measured in water	SoilGrids250m[2]	250 m
25	soil	SANDC	Sand (mass %)	SoilGrids250m[2]	250 m
26	soil	SILTC	Silt (mass %)	SoilGrids250m[2]	250 m
27	soil	AVWAC	Available water capacity (%)	SoilGrids250m[2]	250 m
28	spatial	abs.latit	Absolute		

			latitude		
29	human	HFP	Human footprint	Human Footprint[3]	1 km
30	plant	diversity	Plant diversity	Global plant diversity[4]	5 arc-min
31	plant	npp	Plant productivity	Net primary productivity[5]	5 arc-min

Supplementary Table 4. Comparison of random-forest models running with all 31 predictors (full model) and with the most important predictors (final model).

Soil seed bank	Mean of squared residuals		Variance explained (%)	
	Full model	Final model	Full model	Final model
Diversity	242.46	238.06	44.69	45.69
Density	72248426862	70253049383	65.85	66.79

Supplementary Table 5. Comparison of soil seed bank diversity and density between the Northern and Southern Hemisphere. mean.N, mean value in Northern Hemisphere; mean.S, mean value in Southern Hemisphere; NA, data are not sufficient for t-tests.

Code	Biome	mean.N	mean.S	T	p-value
	**Diversity**				
1	Tropical & Subtropical Moist Broadleaf Forests	25.56	30.30	-1.73	0.09
2	Tropical & Subtropical Dry Broadleaf Forests	14.90	2.89	-	-
3	Tropical & Subtropical Coniferous Forests	37.38	NA	-	-
4	Temperate Broadleaf & Mixed Forests	20.82	25.80	-2.39	0.02
5	Temperate Conifer Forests	21.82	NA	-	-
6	Boreal Forests/Taiga	19.04	NA	-	-
7	Tropical & Subtropical Grasslands, Savannas & Shrublands	17.89	20.64	-1.00	0.32
8	Temperate Grasslands, Savannas & Shrublands	17.26	23.16	-2.80	0.01
9	Flooded Grasslands & Savannas	21.00	14.15	0.92	0.36
10	Montane Grasslands & Shrublands	18.94	17.85	0.43	0.67
11	Tundra	12.11	NA	-	-
12	Mediterranean Forests, Woodlands & Scrub	27.64	23.01	1.98	0.05
13	Deserts & Xeric Shrublands	13.08	19.50	-3.54	0.001
14	Mangroves	20.78	8.10	0.90	0.46
	**Density**				
1	Tropical & Subtropical Moist Broadleaf Forests	14438.04	3801.48	5.19	<0.001
2	Tropical & Subtropical Dry Broadleaf Forests	9188.91	5.02	-	-
3	Tropical & Subtropical Coniferous Forests	6014.91	NA	-	-
4	Temperate Broadleaf & Mixed Forests	15380.31	12475.1	1.08	0.28
5	Temperate Conifer Forests	247063.2	NA	-	-
6	Boreal Forests/Taiga	3768.3	NA	-	-
7	Tropical & Subtropical Grasslands, Savannas & Shrublands	2240.3	6403.7	-2.55	0.01

8	Temperate Grasslands, Savannas & Shrublands	6362.75	11518.6	-3.5	0
9	Flooded Grasslands & Savannas	1640.64	1148.79	0.68	0.5
10	Montane Grasslands & Shrublands	4241.32	1523.41	4.7	0
11	Tundra	2672.2	NA	-	-
12	Mediterranean Forests, Woodlands & Scrub	9786.77	7224.9	2.23	0.03
13	Deserts & Xeric Shrublands	14094.41	7725.67	2.42	0.02
14	Mangroves	3793.34	1028.66	1.45	0.28

Supplementary References:

1. Fick S. E. & Hijmans R. J. Worldclim 2: New 1-km spatial resolution climate surfaces for global land areas. Int. J. Climatol. **37**, 4302–4315 (2017).
2. Hengl, T. et al. SoilGrids250m: Global gridded soil information based on machine learning. PLoS ONE **12**, e0169748 (2017).
3. Venter, O. *et al*. Data from: Global terrestrial Human Footprint maps for 1993 and 2009. Dryad, Dataset. https://doi.org/10.5061/dryad.052q5 (2016).
4. Pausas J. G. & Ribeiro E. Fire and plant diversity at the global scale. Global Ecol. Biogeogr. **26**, 889–897 (2017).
5. Foley J. A., Prentice I. C., Ramankutty N., Levis S., Pollard D., Sitch S. & Haxeltine A. An integrated biosphere model of land surface processes, terrestrial carbon balance and vegetation dynamics. Global Biogeochem. Cycles **10**, 603–628 (1996).
