# Supplementary material for: Global patterns of potential future plant diversity hidden in soil seed banks
Source: Nat Commun. 2021 Dec 2;12:7023. doi: 10.1038/s41467-021-27379-1 (PMC8639999; doi:10.1038/s41467-021-27379-1)
Supplement: Supplementary file 5 — Supplementary Software [file 41467_2021_27379_MOESM5_ESM.zip › Supplementary Software/R codes.docx]

###R codes for analyzing global pattern of soil seed bank###

####################################################

####First, read data from storage device, and name it as “dat1”####

####Codes below show how diversity is analyzed, and density can be analyzed using the same procedure####

#####subset data for diversity and density#####

levels(dat.all$index)

dat1=subset(dat.all, index=="Diversity")

str(dat1) ###6480 obs. for divers, (9218 for density)

##################################################

###get biome for each data point###

library(sp)

library(raster)

library(rgdal)

dat1.sp=SpatialPointsDataFrame(coords = dat1[ , c("longit", "latit")],

data = dat1, proj4string = CRS("+proj=longlat +datum=WGS84 +no_defs +ellps=WGS84 +towgs84=0,0,0"))

###wwf_terr_ecos is the global biome map, which is free available from the World Wildlife Fund website###

biome=readOGR("./global_biomes_geotiff/biome-new/wwf_terr_ecos.shp")

str(biome@data) ##14 variables

biome.ov=over(dat1.sp, biome)

dat1.sp@data$biome=biome.ov$BIOME

biome.name=c("Tropical & Subtropical Moist Broadleaf Forests","Tropical & Subtropical Dry Broadleaf Forests","Tropical & Subtropical Coniferous Forests","Temperate Broadleaf & Mixed Forests","Temperate Conifer Forests","Boreal Forests/Taiga","Tropical & Subtropical Grasslands, Savannas & Shrublands","Temperate Grasslands, Savannas & Shrublands","Flooded Grasslands & Savannas","Montane Grasslands & Shrublands","Tundra","Mediterranean Forests, Woodlands & Scrub","Deserts & Xeric Shrublands","Mangroves")

dat1=dat1.sp@data

####################################################

####excluding (sub-)tropical area from standardization (biome!=(1:3,7))####

####standize seasonal pattern, need to separate North and South####

dat.north=subset(dat1,latit>0&biome!=c("1","2","3","7"))

spring=c(3,4,5)

summer=c(6,7,8)

autumn=c(9,10,11)

winter=c(12,1,2)

dat.spr=subset(dat.north,Time.sample%in%spring)

dat.sum=subset(dat.north,Time.sample%in%summer)

dat.aut=subset(dat.north,Time.sample%in%autumn)

dat.win=subset(dat.north,Time.sample%in%winter)

dat.south=subset(dat1,latit<0&biome!=c("1","2","3","7"))

spring=c(9,10,11)

summer=c(12,1,2)

autumn=c(3,4,5)

winter=c(6,7,8)

dat.spr1=subset(dat.south,Time.sample%in%spring)

dat.sum1=subset(dat.south,Time.sample%in%summer)

dat.aut1=subset(dat.south,Time.sample%in%autumn)

dat.win1=subset(dat.south,Time.sample%in%winter)

dat.spr=rbind(dat.spr, dat.spr1)

dat.sum=rbind(dat.sum, dat.sum1)

dat.aut=rbind(dat.aut, dat.aut1)

dat.win=rbind(dat.win, dat.win1)

dat.spr$season=c("spring")

dat.sum$season=c("summer")

dat.aut$season=c("autumn")

dat.win$season=c("winter")

m.spr=mean(dat.spr$Value)

m.sum=mean(dat.sum$Value)

m.aut=mean(dat.aut$Value)

m.win=mean(dat.win$Value)

dat.season=rbind(dat.spr,dat.sum,dat.aut, dat.win)

dat.season$season=factor(dat.season$season)

######plot seasonal patterns########

library(ggplot2)

library(ggsci)

col=pal_npg("nrc")(9)

p.season=ggplot(data=dat.season, aes(x=factor(season, levels=c("spring","summer","autumn","winter")), y=Value, fill=season)) +

geom_violin(width=0.75, trim=F, show.legend=F) +

geom_boxplot(width=0.1, outlier.alpha = 0.01, fill="white",show.legend=F) +

annotate("rect", xmin = .5, xmax = 4.5, ymin = 14000, ymax =15000, fill = "white") +

annotate("text", x=c(1:4), y=c(rep(15000,4)), label=c(round(m.spr,2),round(m.sum,2),round(m.aut,2),round(m.win,2)), size=3) +

ylab("Diversity") + xlab("Season") +

ylim(0,15000)+

theme_bw() +theme(panel.grid.major = element_blank(), panel.grid.minor = element_blank()) +

theme(axis.text.x= element_text(size=8)) + theme(axis.text.y = element_text(size=8)) +

theme(axis.title.x = element_text(size=8), axis.title.y = element_text(size=8))

p.season ##view plot

######winter has the highest value, standardize other season########

for (i in (1:length(dat.season$season))){

if (dat.season$season[i]=="spring"){

dat.season$Value[i]=dat.season$Value[i]*m.win/m.spr

}

else

if (dat.season$season[i]=="summer"){

dat.season$Value[i]=dat.season$Value[i]*m.win/m.sum

}

else

if (dat.season$season[i]=="autumn"){

dat.season$Value[i]=dat.season$Value[i]*m.win/m.aut

}

else FALSE

}

######write back values to dat1########

m=match(dat1$X, dat.season$X) ##X is the rowname

for (i in (1:length(dat1$X))){

if (!is.na(m[i])){

dat1$Value[i]=dat.season$Value[m[i]]

}

else FALSE

}

####################################################

######remove outliers before analysis#############

library(EnvStats)

rosner=rosnerTest(dat1$Value, k = length(boxplot(dat1$Value)$out), alpha = 0.05, warn = TRUE)

Outlier=subset(rosner$all.stats,Outlier=="TRUE")

Outlier$Obs.Num

caps = quantile(dat1$Value, probs=c(.05, .95), na.rm = T)

dat1$Value[Outlier$Obs.Num] = caps[2] ###data that lie above the upper limit, with the value of 95th%ile

mean(dat1$Value)

####################################################

####standardize area, not for density####

dat1$stand.area=gsub("m2","",dat1$Area)

dat1$stand.area=as.numeric(as.character(dat1$stand.area))

which(is.na(dat1$stand.area),arr.ind=T)

######plot frequency distribution########

tab.area=data.frame(table(dat1$Area))

tab.area[order(tab.area$Freq), ] ## highest frequency: 0.01m2 779

p.area.fr=ggplot(data=dat1, aes(x=Area, fill=Value)) +

geom_bar(stat = "count") +

annotate("text", x = 320, y = 800, label = "0.01m^2: 779 cases", size=3) +

xlab("Sampling area (m^2)")+ylab("Count") +

theme_bw() +theme(panel.grid.major = element_blank(), panel.grid.minor = element_blank()) +

theme(axis.text.x = element_blank(), axis.ticks.x = element_blank(), legend.position="none")

p.area.fr ###view plot

####fitting species-area curve: S = CA^z -> log10S = log10 C + z log10 A####

####model relationships in different biomes####

biome.lel=levels(dat1$biome)

intercept=c()

slope=c()

p.value=c()

dat2=c()

for (i in 1:length(biome.lel)){

dat.biome=subset(dat1,biome==biome.lel[i])

dat1.area=subset(dat.biome, stand.area<10) ##excluding extreme high area

m.area=lm(log10(Value+0.000001)~log10(stand.area), dat1.area)

summary(m.area)

intercept=c(intercept, coef(m.area)[1])

slope=c(slope, coef(m.area)[2])

p.value=c(p.value,summary(m.area)$coefficients[,"Pr(>|t|)"][2])

###standardize to 0.01m2###

dat.biome$stand.Value=dat.biome$Value*dat.biome$stand.area^coef(m.area)[2]/0.01^coef(m.area)[2]

summary(dat.biome$stand.Value)

dat2=rbind(dat2,dat.biome)

}

area.reg=data.frame(biome=c(biome.name), intercept=intercept, slope=slope, p.value=p.value)

area.reg[,2:4]=round(area.reg[,2:4],3)

area.reg

######write values back to dat1########

m=match(dat1$X, dat2$X) ##X is the rowname

for (i in (1:length(dat1$X))){

if (!is.na(m[i])){

dat1$stand.Value[i]=dat2$stand.Value[m[i]]

}

else FALSE

}

####################################################

####standardize depth####

###for density, not standardized for area (16 variables): dat1$stand.Value =dat1$Value; dat1$stand.area=dat1$Area###

tab.depth=data.frame(table(dat1$Depth))

tab.depth[order(tab.depth$Freq), ] ## highest frequency: 0-5cm 2348

p.dep.fr=ggplot(data=dat1, aes(x=Depth, fill=Value)) +

geom_bar(stat = "count") +

theme(axis.text.x = element_text(angle = 90)) +

annotate("text", x = 60, y = 2500, label = "0-5cm: 2348 cases") +

xlab("Sampling depth (cm)")+ylab("Count") +

theme_bw() +theme(panel.grid.major = element_blank(), panel.grid.minor = element_blank()) +

theme(axis.text.x = element_blank(), axis.ticks.x = element_blank(), legend.position="none")

p.dep.fr

####change values to range from 0####

library(strex)

dat1$upper=str_nth_number(as.character(dat1$Depth), n=1, decimals = TRUE) ##extract first number in Depth

dat1$lower=str_nth_number(as.character(dat1$Depth), n=2, decimals = TRUE) ##extract second number in Depth

dat1$lower[is.na(dat1$lower)]=0 ##surface (0cm) did not have the lower

dat1$dep=dat1$lower-dat1$upper

####model depth relationships in different biomes####

intercept=c()

slope.upper=c()

slope.lower=c()

p.value=c()

dat2=c()

for (i in 1:length(biome.lel)){

dat.biome=subset(dat1,biome==biome.lel[i])

m.dep=lm(log10(stand.Value+0.000001)~log10(upper+0.000001)+log10(lower+0.000001), dat.biome)

summary(m.dep)

intercept=c(intercept, coef(m.dep)[1])

slope.upper=c(slope.upper,coef(m.dep)[2])

slope.lower=c(slope.lower,coef(m.dep)[3])

p.value=c(p.value,summary(m.dep)$r.squared)

###standardize to 0-5cm: log(D05)-log(Dul)=log(c)-log(c)+z1*(log(D0)-log(Du))+z2*(log(D5)-log(Dl))###

for (j in (1:length(dat.biome$Source))){

if (dat.biome$stand.Value[j]==0){ ##0 need not to standardize

dat.biome$stand.Value0[j]=dat.biome$stand.Value[j]

}

else{

if (dat.biome$Depth[j]=="0-5cm"){

dat.biome$stand.Value0[j]=dat.biome$stand.Value[j]

}

else{

dat.biome$stand.Value0[j]=dat.biome$stand.Value[j]*((0+0.000001)/(dat1$upper[j]+0.000001))^coef(m.dep)[2] * ((5+0.000001)/(dat1$lower[j]+0.000001)) ^coef(m.dep)[3]

}

}

}

summary(dat.biome$stand.Value0)

dat2=rbind(dat2,dat.biome)

}

dep.reg=data.frame(biome=c(biome.name), intercept=intercept, slope.upper=slope.upper,slope.lower=slope.lower, adj.p.value=p.value)

dep.reg[,2:5]=round(dep.reg[,2:5],3)

dep.reg

######write values back to dat1########

m=match(dat1$X, dat2$X) ##X is the rowname

for (i in (1:length(dat1$X))){

if (!is.na(m[i])){

dat1$stand.Value0[i]=dat2$stand.Value0[m[i]]

}

else FALSE

}

##################################################

###compare North and South Hemisphere###

North=subset(dat1,latit>0);North$Hemisphere=c("Northern")

South=subset(dat1,latit<0);South$Hemisphere=c("Southern")

dat.s.n=rbind(North,South)

mean.N=c();mean.S=c()

t.statistic=c()

p.value=c()

for (i in 1:length(biome.lel)){

dat.biome=subset(dat.s.n,biome==biome.lel[i])

if (sum(dat.biome$Hemisphere=="Northern")<3 | sum(dat.biome$Hemisphere=="Southern")<3){

hemis.t$statistic=c("NA");hemis.t$p.value=c("NA")

} else {

hemis.t=t.test(dat.biome$stand.Value0[dat.biome$Hemisphere=="Northern"], y=dat.biome$stand.Value0[dat.biome$Hemisphere=="Southern"])

}

mean.N=c(mean.N,mean(dat.biome$stand.Value0[dat.biome$Hemisphere=="Northern"],na.rm=T));mean.S=c(mean.S,mean(dat.biome$stand.Value0[dat.biome$Hemisphere=="Southern"],na.rm=T))

t.statistic=c(t.statistic,hemis.t$statistic)

p.value=c(p.value,hemis.t$p.value)

}

sn.reg=data.frame(biome=c(biome.name), mean.Northern=mean.N, mean.Southern=mean.S, t.statistic=t.statistic, p.value=p.value)

sn.reg

###############difference between biomes####################

biome=levels(dat1$biome)

mean=aggregate(dat1$stand.Value0, list(dat1$biome), FUN=mean)

mean=mean[order(mean$x),]

###because data are non-normal, using Kruskal-Wallis test to detect differences between biomes######

kruskal.test(stand.Value0~biome, data=dat1)

p.test=pairwise.wilcox.test(dat1$stand.Value0, reorder(dat1$biome, -dat1$stand.Value0), p.adjust.method = "BH")

p.test$p.value

###convert to standard matrix that can be used by multcomView####

library(multcompView)

tri.to.squ<-function(x)

{

rn<-row.names(x)

cn<-colnames(x)

an<-unique(c(cn,rn))

myval<-x[!is.na(x)]

mymat<-matrix(1,nrow=length(an),ncol=length(an),dimnames=list(an,an))

for(ext in 1:length(cn))

{

for(int in 1:length(rn))

{

if(is.na(x[row.names(x)==rn[int],colnames(x)==cn[ext]])) next

mymat[row.names(mymat)==rn[int],colnames(mymat)==cn[ext]]<-x[row.names(x)==rn[int],colnames(x)==cn[ext]]

mymat[row.names(mymat)==cn[ext],colnames(mymat)==rn[int]]<-x[row.names(x)==rn[int],colnames(x)==cn[ext]]

}

}

return(mymat)

}

mymat=tri.to.squ(p.test$p.value)

myletters=multcompLetters(mymat,compare="<=", threshold=0.05, Letters=letters)

p.biome=ggplot(data=dat1, aes(x=factor(dat1$biome, levels = paste(mean$Group.1)), y=stand.Value0, fill=biome)) +

geom_violin(width=1.0, trim=F, show.legend=F) +

geom_boxplot(width=0.1, outlier.alpha = 0.01, fill="white",show.legend=F) +

annotate("rect", xmin = .5, xmax = 14.5, ymin = 7000, ymax =8000, fill = "white") +

annotate("text", x=c(1:14), y=rep(8000,14), label=round(mean$x,0), size=1.8) +

annotate("text", x=c(rownames(data.frame(myletters$Letters))), y=7500, label=c(unname(myletters$Letters)), col="black", size=1.8)+

ylim(0,8000)+

ylab("Diversity") + xlab("Biome") +

theme_bw() +theme(panel.grid.major = element_blank(), panel.grid.minor = element_blank()) +

theme(axis.text.x= element_text(size=8,angle=0)) + theme(axis.text.y = element_text(size=8)) +

theme(axis.title.x = element_text(size=8), axis.title.y = element_text(size=8)) +

scale_x_discrete(limits =paste(mean$Group.1))

p.biome ##view plot

##########################################################

###check spatial autocorrelation in data###

library(geoR)

data.geoR = as.geodata(dat1, coords.col = 5:6, data.col = 20)

summary(data.geoR)

plot.geodata(data.geoR)

## Define the variogram binning (100 regular distance bins)

test = dist(dat1[,c("latit", "longit")])

library(Rcmdr)

bla = bin.var(test, bins = 100, method = c("proportions"),labels = NULL)

dtmp = c()

for (i in 1:length(levels(bla)))

{

if(i==1){

lev = as.character(levels(bla)[i])

tmp1 = strsplit(lev, "[[]")[[1]][2]

bound1 = as.numeric(strsplit(tmp1, ",")[[1]][1])

tmp2 = strsplit(tmp1, ",")[[1]][2]

bound2 = as.numeric(strsplit(tmp2, "[]]")[[1]][1])

dtmp = c(dtmp, bound1+(bound2-bound1)/2)

}

if(i!=1){

lev = as.character(levels(bla)[i])

tmp1 = strsplit(lev, "[(]")[[1]][2]

bound1 = as.numeric(strsplit(tmp1, ",")[[1]][1])

tmp2 = strsplit(tmp1, ",")[[1]][2]

bound2 = as.numeric(strsplit(tmp2, "[]]")[[1]][1])

dtmp = c(dtmp, bound1+(bound2-bound1)/2)

}

}

# Computes sample (empirical) variogram, including its envelop (obtained by permutation)

bin = variog(data.geoR, uvec = dtmp)

env = variog.mc.env(data.geoR, obj.variog = bin)

plot(bin, envelope = env, pch=21, bg="grey", xlab="Distance (km)", ylab="Semivariance", cex=1.5, cex.lab=1.5, cex.axis=1.4)

##########################################################

###################geostatical analyses################

dat1.sp=c()

dat1.sp=SpatialPointsDataFrame(coords = dat1[ , c("longit", "latit")],

data = dat1, proj4string = CRS("+proj=longlat +datum=WGS84 +no_defs +ellps=WGS84 +towgs84=0,0,0"))

###view points####

library(rworldmap)

library(rworldxtra) ##need for "high" resolution

wmap = getMap(resolution = "low")

mapDevice()

plot(wmap, border="grey40", col="grey80")

points(dat1.sp,pch=19,col=col[8],cex=0.6)

rect(xleft=-180, xright=179.99, ybottom=-90, ytop=85.9996)

rm(wmap)

###############################################

####read environmental variables, which need to be downloaded from different source (see Supplementary Tables). Below give an example to read them (bioclim) to R###

climate variable####

fpath=c()

climate=readGDAL("./wc2.0_5m_bio/wc2.0_bio_5m_1.tif")

for (i in (2:19)){

fpath[i]=paste("./wc2.0_5m_bio/wc2.0_bio_5m_",i,".tif",sep="")

cli= readGDAL(fpath[i])$band1

climate@data=cbind(climate@data,cli)

}

colnames(climate@data)=c("AMT","TDR","Isoth","Tseason","TWM","TCM","ATR","TWEQ","TDQ","TWQ","TCQ","AP","PWM","PDM","Pseason","PWeQ","PDQ","PWQ","PCQ")

str(climate)

###After all variable have been read, need to combine them to single database. Below is an example###

environ=climate

soil1=over(environ,soil)

environ@data=cbind(environ@data,soil1)

environ@data$pH=environ@data$pH/10

str(environ) ##view environment

####################################################

#############crop environments using dat1.sp#############

dat.en = over(dat1.sp,environ)

dat1.sp@data=cbind(dat1.sp@data,dat.en)

dat.rf=cbind(dat1.sp@data[,5],dat1.sp@data[,21:51]) ##5 are latit, [19:45] for density (stand.area is not present)

colnames(dat.rf)[1]=c("abs.latit"); dat.rf$abs.latit=abs(dat.rf$abs.latit) ###change to absolute latitude

#############random forest#############

library(randomForest)

#######try ntree############

rf_ntree = randomForest(stand.Value0 ~., data=dat.rf, na.action=na.omit)

plot(rf_ntree)

########try mtry###########

dat.sel=na.omit(dat.rf)

mtry=tuneRF(x=cbind(dat.sel[,1],dat.sel[,3:32]), y=dat.sel[,2], ntreeTry=100, plot=TRUE, doBest=T)

mtry=mtry$mtry

######################

set.seed(123)

m.rf = randomForest(stand.Value0 ~., data=dat.rf, na.action=na.omit, ntree=100, mtry=mtry, proximity=TRUE, importance=TRUE)

print(m.rf)

imp=data.frame(importance(m.rf))

varImpPlot(m.rf)

###construct a table for variable class#####

variable=data.frame(colnames(dat.rf[-2])); colnames(variable)=c("variable")

class=data.frame(c(rep("spatial",1),rep("climate",19),rep("soil",8), rep("human",1),rep("plant",2))); colnames(class)=c("class")

tab.class=cbind(variable,class)

tab.class=cbind(imp,tab.class)

tab.class$number=c(1:31)

####plot importance######

p.IncNode=ggplot(data=tab.class, aes(x=reorder(variable,IncNodePurity), y=IncNodePurity, fill=class)) +

geom_bar(stat="identity", color="black") +

ylab("Increase in node purity") +

coord_flip() +

theme_bw() +theme(panel.grid.major = element_blank(), panel.grid.minor = element_blank()) +

theme(axis.title.y=element_blank(), axis.title.x=element_text(size=5, face="bold"))+

theme(axis.text.x = element_text(size=5, face="bold", color="black"), axis.text.y = element_text(size=5, face="bold", color="black"))+

theme( legend.title = element_text(size = 8), legend.text = element_text(size = 8) ) +

scale_fill_manual(values = c(col[2],col[5],col[7],col[9],col[1]))+

theme(legend.position = c(.8,.2))

p.IncNode ##view plot

p.IncMSE=ggplot(data=tab.class, aes(x=reorder(variable,X.IncMSE), y=X.IncMSE, fill=class)) +

geom_bar(stat="identity", color="black") +

ylab("Increase in MSE") +

coord_flip() +

theme_bw() +theme(panel.grid.major = element_blank(), panel.grid.minor = element_blank()) +

theme(axis.title.y=element_blank(), axis.title.x=element_text(size=5, face="bold"))+

theme(axis.text.x = element_text(size=5, face="bold", color="black"), axis.text.y = element_text(size=5, face="bold", color="black"))+

theme( legend.title = element_text(size = 8), legend.text = element_text(size = 8) ) +

scale_fill_manual(values = c(col[2],col[5],col[7],col[9],col[1]))+

theme(legend.position = c(.8,.2))

p.IncMSE ##view plot

#####Variable selection###

library(VSURF)

set.seed(123)

var.sel = VSURF(stand.Value0 ~., data=dat.rf, na.action=na.omit)

summary(var.sel)

dev.new(height=8,width=10)

plot(var.sel, step = "pred", imp.sd = FALSE, var.names = TRUE, cex.lab=1.4)

var.prd=colnames(dat.rf[-2])[var.sel$varselect.pred]

var.prd

#####select the most important using var.prd###

dat.rf.sel=dat.sel[,var.prd]

dat.rf.sel=data.frame(stand.Value0=dat.sel$stand.Value0, dat.rf.sel)

#######try ntree############

rf_ntree = randomForest(stand.Value0 ~., data=dat.rf.sel)

plot(rf_ntree)

########try mtry###########

mtry=tuneRF(x=dat.rf.sel[,2:6], y=dat.rf.sel[,1], ntreeTry=100, plot=TRUE, doBest=T) ##2:10 for diversity

mtry=mtry$mtry

set.seed(123)

m.sel = randomForest(stand.Value0 ~., data=dat.rf.sel, na.action=na.omit, ntree=100, mtry=mtry, proximity=TRUE, importance=TRUE, keep.inbag = T, keep.forest = T)

print(m.sel)

imp.sel=importance(m.sel)

imp.sel

varImpPlot(m.sel)

#######crossValidation############

library(rfUtilities)

rf.cv=rf.crossValidation(m.sel, xdata=dat.rf.sel[,2:6], p = 0.1, n = 99) ##2:10 for diversity

dev.new(width=10, height=7.5)

plot(rf.cv, stat = "var.exp", xlab="Bootstrap iteration", ylab="Variance explained", main=NULL, cex.lab=1.4, cex.axis=1.2)

#######partial plot############

library(forestFloor)

set.seed(123)

ff = forestFloor(m.sel, X=dat.rf.sel[2:6], binary_reg = F, calc_np=F)

Col = fcol(ff,cols=1)

###check sequence, Col###

dev.new()

plot(ff, col=Col)

imp.sel=data.frame(imp.sel)

imp.MSE=imp.sel[order(-imp.sel$X.IncMSE),]

imp.Node=imp.sel[order(-imp.sel$IncNodePurity),]

match(imp.Node$IncNodePurity, imp.MSE$IncNodePurity)

plot(ff, col=alpha(col[6], 0.6), plot_seq=c(match(imp.Node$IncNodePurity, imp.MSE$IncNodePurity)), plot_GOF = T, GOF_args = list(col=col[8]), pch = 19, cex=1.2, cex.axis=1.2, main=NULL, mfrow=c(3,3))

############################################################

######global predition############

environ@data$latit=coordinates(environ)[,2]

environ@data$longit=coordinates(environ)[,1]

environ@data$abs.latit=abs(environ@data$latit) ##change to absolute latitude

environ@data$rownames=rownames(environ@data)

newdata=environ@data

dim(newdata)

newdata=na.exclude(newdata)

dim(newdata)

rownames=newdata$rownames

rownames=data.frame(rownames)

newdata=newdata[,xlab]

dim(newdata)

prd.glob=predict(m.sel, newdata = newdata, type="response")

prd.glob=data.frame(prd.glob)

prd.glob$rownames=rownames$rownames

m = match(environ@data$rownames, prd.glob$rownames)

str(m)

environ@data$prd.Value =prd.glob$prd.glob[m]

length(na.exclude(environ@data$prd.Value))

range(na.exclude(environ@data$prd.Value))

library(rgeos)

wmap = getMap(resolution = "low")

wmap = gUnaryUnion(wmap) ##merge country boundary

library(spatial.tools)

env.bbox = bbox(environ)

wmap.s=bbox_to_SpatialPolygons(env.bbox, CRS("+proj=longlat +ellps=WGS84 +datum=WGS84 +no_defs"))

wmap.env=gIntersection(wmap.s, wmap)

####mapping###

library(colormap)

col=c(colormap(colormap=colormaps$portland, nshades=100, reverse = F))

col1=c(rep(col[1:30],each=3),col[31:100])

mapDevice()

spplot(environ1, "prd.Value", sp.layout=list(wmap.env, first = FALSE, col = 'grey'), col.regions = col1, colorkey = list(space = "bottom", height = 0.6, width = 0.9, labels=list(at = c(0,2,4,6),limits=c(0,8), cex=1)))
