# Supplementary material for: Global patterns of potential future plant diversity hidden in soil seed banks
Source: Nat Commun. 2021 Dec 2;12:7023. doi: 10.1038/s41467-021-27379-1 (PMC8639999; doi:10.1038/s41467-021-27379-1)
Supplement: Supplementary file 6 — Reporting Summary [file 41467_2021_27379_MOESM6_ESM.pdf]

nature portfolio

Corresponding author(s): Zhenying Huang

Last updated by author(s): Nov 12, 2021

Reporting Summary

Nature Portfolio wishes to improve the reproducibility of the work that we publish. This form provides structure for consistency and transparency in reporting. For further information on Nature Portfolio policies, see our Editorial Policies and the Editorial Policy Checklist.

Statistics

For all statistical analyses, confirm that the following items are present in the figure legend, table legend, main text, or Methods section.

n/a	Confirmed	
☐	☒	The exact sample size (*n*) for each experimental group/condition, given as a discrete number and unit of measurement
☒	☐	A statement on whether measurements were taken from distinct samples or whether the same sample was measured repeatedly
☐	☒	The statistical test(s) used AND whether they are one- or two-sided<br>*Only common tests should be described solely by name; describe more complex techniques in the Methods section.*
☒	☐	A description of all covariates tested
☐	☒	A description of any assumptions or corrections, such as tests of normality and adjustment for multiple comparisons
☐	☒	A full description of the statistical parameters including central tendency (e.g. means) or other basic estimates (e.g. regression coefficient) AND variation (e.g. standard deviation) or associated estimates of uncertainty (e.g. confidence intervals)
☐	☒	For null hypothesis testing, the test statistic (e.g. *F*, *t*, *r*) with confidence intervals, effect sizes, degrees of freedom and *P* value noted<br>*Give P values as exact values whenever suitable.*
☒	☐	For Bayesian analysis, information on the choice of priors and Markov chain Monte Carlo settings
☒	☐	For hierarchical and complex designs, identification of the appropriate level for tests and full reporting of outcomes
☒	☐	Estimates of effect sizes (e.g. Cohen's *d*, Pearson's *r*), indicating how they were calculated

Our web collection on statistics for biologists contains articles on many of the points above.

Software and code

Policy information about availability of computer code

Data collection	R (version 3.4.3)
Data analysis	R (version 3.4.3), EnvStats package (version 2.4.0), randomForest package (version 4.6-14), VSURF package (version 1.1.0), forestFloor package (version 1.11.1), rfUtilities package (version 2.1-4).

For manuscripts utilizing custom algorithms or software that are central to the research but not yet described in published literature, software must be made available to editors and reviewers. We strongly encourage code deposition in a community repository (e.g. GitHub). See the Nature Portfolio guidelines for submitting code & software for further information.

Data

Policy information about availability of data

All manuscripts must include a data availability statement. This statement should provide the following information, where applicable:

- Accession codes, unique identifiers, or web links for publicly available datasets
- A description of any restrictions on data availability
- For clinical datasets or third party data, please ensure that the statement adheres to our policy

Data and references from which data were collected supporting the findings of this study are available in the Supplementary Data 1 & 2. Terrestrial Ecoregions of the World (TEOW) are publicly available on the World Wildlife Fund (WWF) website [https://www.worldwildlife.org/]. Climate data reported in this study are publicly available on the WorldClim database [https://www.worldclim.org/]. Soil data are publicly available on the SoilGrids system [https://soilgrids.org/].

Field-specific reporting

Please select the one below that is the best fit for your research. If you are not sure, read the appropriate sections before making your selection.

☐ Life sciences ☐ Behavioural & social sciences ☒ Ecological, evolutionary & environmental sciences

For a reference copy of the document with all sections, see nature.com/documents/nr-reporting-summary-flat.pdf

Ecological, evolutionary & environmental sciences study design

All studies must disclose on these points even when the disclosure is negative.

Study description	Our study extracted data from published literature, no new data have been assembled. We compiled a global dataset comprising 15,698 records of species diversity and density for soil seed banks in natural plant communities worldwide to quantify their environmental determinants and global patterns. The random-forest algorithm was used to model the relationships between predictors and soil seed banks, which is a data-driven ensemble learning approach that averages over multiple regression trees, each of which uses a random subset of all the model variables to predict a response.
Research sample	We built a global dataset comprising 15,698 published records for soil seed banks worldwide, derived from 1502 publications.
Sampling strategy	Search and selection criteria for our synthesis were described in detail in method section.
Data collection	X.Y. and R.G. collected the data. We read each published literature, extracted data from the text, tables, digitized graphs or supplementary materials and recorded data in Excel.
Timing and spatial scale	Timing and spatial scale was defined by the authors of the 1502 published studies.
Data exclusions	No data were excluded.
Reproducibility	To test the sensitivity of partial model performance, we performed K-fold cross-validations that test the sensitivity of model predictions to the exclusion of random subsets from the training data.
Randomization	No randomization procedure was needed because we used published literature and no new data have been assembled.
Blinding	No blinding was needed because we used published literature.

Did the study involve field work? ☐ Yes ☒ No

Reporting for specific materials, systems and methods

We require information from authors about some types of materials, experimental systems and methods used in many studies. Here, indicate whether each material, system or method listed is relevant to your study. If you are not sure if a list item applies to your research, read the appropriate section before selecting a response.

Materials & experimental systems

n/a	Involved in the study	
☒	☐	Antibodies
☒	☐	Eukaryotic cell lines
☒	☐	Palaeontology and archaeology
☒	☐	Animals and other organisms
☒	☐	Human research participants
☒	☐	Clinical data
☒	☐	Dual use research of concern

Methods

n/a	Involved in the study	
☒	☐	ChIP-seq
☒	☐	Flow cytometry
☒	☐	MRI-based neuroimaging
